# Digitizing a Face-to-Face Group Fatigue Management Program: Exploring the Views of People With Multiple Sclerosis and Health Care Professionals Via Consultation Groups and Interviews

**DOI:** 10.2196/10951

**Published:** 2019-05-22

**Authors:** Sarah Thomas, Andy Pulman, Peter Thomas, Sarah Collard, Nan Jiang, Huseyin Dogan, Angela Davies Smith, Susan Hourihan, Fiona Roberts, Paula Kersten, Keith Pretty, Jessica K Miller, Kirsty Stanley, Marie-Claire Gay

**Affiliations:** 1 Bournemouth University Clinical Research Unit Faculty of Health & Social Sciences Bournemouth University Bournemouth United Kingdom; 2 Faculty of Science & Technology Department of Computing and Informatics Bournemouth University Bournemouth United Kingdom; 3 Bristol and Avon Multiple Sclerosis Service North Bristol National Health Service Trust Bristol United Kingdom; 4 Therapy and Rehabilitation Service National Hospital for Neurology and Neurosurgery University College London Hospitals London United Kingdom; 5 Long Term Conditions Team The Walton Centre National Health Service Trust Liverpool United Kingdom; 6 School of Health Sciences University of Brighton Brighton United Kingdom; 7 Department of Sociology University of Cambridge Cambridge United Kingdom; 8 Dorset Multiple Sclerosis Service Poole Hospital National Health Service Foundation Trust Poole United Kingdom; 9 Paris Nanterre University Paris France

**Keywords:** multiple sclerosis, fatigue, telemedicine, mobile health, FACETS, fatigue management

## Abstract

**Background:**

Fatigue is one of the most common and debilitating symptoms of multiple sclerosis (MS) and is the main reason why people with MS stop working early. The MS Society in the United Kingdom funded a randomized controlled trial of *FACETS*—a face-to-face group-based fatigue management program for people with multiple sclerosis (pwMS)—developed by members of the research team. Given the favorable trial results and to help with implementation, the MS Society supported the design and printing of the FACETS manual and materials and the national delivery of FACETS training courses (designed by the research team) for health care professionals (HCPs). By 2015 more than 1500 pwMS had received the FACETS program, but it is not available in all areas and a face-to-face format may not be suitable for, or appeal to, everyone. For these reasons, the MS Society funded a consultation to explore an alternative Web-based model of service delivery.

**Objective:**

The aim of this study was to gather views about a Web-based model of service delivery from HCPs who had delivered FACETS and from pwMS who had attended FACETS.

**Methods:**

Telephone consultations were undertaken with FACETS-trained HCPs who had experience of delivering FACETS (*n=8*). Three face-to-face consultation groups were held with pwMS who had attended the FACETS program: London (n=4), Liverpool (n=4), and Bristol (n=7). The interviews and consultation groups were digitally recorded and transcribed. A thematic analysis was undertaken to identify key themes. Toward the end of the study, a *roundtable* meeting was held to discuss outcomes from the consultation with representatives from the MS Society, HCPs, and pwMS.

**Results:**

Key challenges and opportunities of designing and delivering an integrated Web-based version of FACETS and maintaining user engagement were identified across 7 themes (delivery, online delivery, design, group, engagement, interactivity, and HCP relationships). Particularly of interest were themes related to replicating the group dynamics and the lack of high-quality solutions that would support the FACETS’ weekly homework tasks and symptom monitoring and management.

**Conclusions:**

A minimum viable Web-based version of FACETS was suggested as the best starting point for a phased implementation, enabling a solution that could then be added to over time. It was also proposed that a separate study should look to create a free stand-alone digital toolkit focusing on the homework elements of FACETS. This study has commenced with a first version of the toolkit in development involving pwMS throughout the design and build stages to ensure a user-centered solution.

## Introduction

**Background**

Multiple sclerosis (MS) is a neurological condition affecting the central nervous system. Over 2.5 million people have MS worldwide [1]. Fatigue is one of the most common and debilitating symptoms of MS and is the main reason why people with MS (pwMS) stop working early. Its invisible nature can make it difficult to understand and recognize. There are around 10,000 unique visits to the UK MS Society’s fatigue Web page [2] each year. Fatigue management is one of the most common helpline enquiry topics and the most frequently accessed, printed, and downloaded resource material. Fatigue is the third priority in the James Lind Alliance research priorities for MS [3].

MS is typically diagnosed when people are at their most productive age (average onset at 29 years of age) [1] and the burden on the global economy is increasing as costs have shifted toward outpatient care since the mid-1990s [4]. Against a backdrop of a restrained health care environment and increased emphasis on self-management, digital solutions offer huge potential to provide personalized and cost-effective ways of improving aspects of health and social care [5-6].

FACETS (Fatigue: Applying Cognitive behavioural and Energy effectiveness Techniques to lifeStyle) is a group-based fatigue management program for pwMS, developed by a team based at Bournemouth University and Poole Hospital. The FACETS program blends energy effectiveness principles with cognitive behavioral (CB) approaches [7,8] and consists of 6, weekly, face-to-face (F2F) sessions (Table 1). It is designed to be delivered by 2 health care professionals (HCPs) in small groups (6 to 10 people). Each session incorporates brief presentations, group discussions, and activities. Attendees are asked to complete weekly homework tasks to try out strategies covered in sessions.

The MS Society funded a national multi-center pragmatic randomized controlled trial (RCT) of FACETS versus usual care. Findings from the RCT (n=164) indicated that FACETS was effective [9] with significant improvements in fatigue self-efficacy (Standardized Effect Size [SES]=.36) and fatigue severity (SES=−.35) at 4 months follow-up in the FACETS arm relative to the usual care arm, with most participants reporting they had successfully implemented fatigue management strategies [10]. These improvements largely persisted a year on from the FACETS program (SES fatigue self-efficacy=−.29 and SES fatigue severity=.34), and there was also a significant improvement in MS-specific quality of life (SES=−.24) that had not been present at 4 months [11]. Given these favorable results, and to help with FACETS implementation, the MS Society supported the design and printing of the facilitator manual and participant materials and the national delivery of 1-day training courses (designed by members of the research team) for HCPs. By 2015, around 200 HCPs had been trained to deliver FACETS and an estimated 1500 pwMS in the United Kingdom had received the FACETS program [12].

### Rationale and Aims

The FACETS program is not available in all areas of the United Kingdom. Work commitments, mobility or cognitive impairments, rurality and transport issues, or personal preferences might mean digital delivery would be more convenient or appealing for some pwMS. The MS Society was therefore keen to consider alternative delivery models. The project aims were to undertake a consultation to:

1. Gather views from HCPs and pwMS about a Web-based model of service delivery, considering aspects of delivery format and mode.
2. Obtain feedback about how best to adapt FACETS for Web-based delivery (known as *cFACETS*).

**Table 1:** Overview of the FACETS program.

Session number	Session title	Homework element(s)
1	What is MS^a^-related fatigue?	Activity/fatigue diary; Energy measure
2	Opening an *energy account*	Rest/activity/sleep planner
3	Budgeting energy and *smartening up* goals	Goal-setting exercise
4	The stress response; the cognitive behavioral model	Fatigue thought diary
5	Putting unhelpful thoughts on trial	Thought challenge sheet
6	Recapping and taking the program forward	*Keeping on Track* planner

^a^MS: multiple sclerosis.

## Methods

Ethical approval was obtained from Bournemouth University (ref. 14371).

### Study Design

We used a combination of telephone interviews with health care professionals and consultation groups with people with MS. Toward the end of the project, we held a roundtable meeting in London to discuss the findings from the consultation with representatives from the MS Society, HCPs, and pwMS.

### Participants, Recruitment, and Consent

We aimed to conduct 8 telephone interviews with HCPs. HCPs were identified via the project lead’s networks and via an MS Society database. They were sent an information sheet and a copy of the consent form via email. Before the interview, the consultation coordinator answered any questions they had, checked whether they were willing to be audio-recorded, and explained the consent process. If they wished to take part, their verbal consent was recorded at the start of the telephone consultation. If they preferred not to be audio-recorded, they were given the option of providing written consent, with notes taken instead.

We planned to conduct consultation groups with pwMS in 3 UK locations (London, Bristol, and Liverpool). PwMS were identified via a gatekeeper (these were HCPs) at each location. The gatekeeper identified pwMS who had previously attended the FACETS program (Bristol and London), those attending MS clinics (London and Liverpool), or those currently attending a FACETS program (all 3 locations). They gave or sent them information about the service improvement consultation and a copy of the consent form. Those interested in participating were asked to email or telephone the consultation coordinator. Before the consultation group, the consultation coordinator answered any questions they had, checked if they were willing to be audio-recorded, and explained the consent process. If they preferred not to be audio-recorded, they were offered a one-to-one telephone interview with notes taken instead.

### Procedures and Measures

The consultation groups and interviews were undertaken by an experienced qualitative researcher. Topic guides were used to ensure areas of interest (such as aspects related to delivery mode and format) were covered, while still allowing flexibility. The telephone interviews with HCPs were audio-recorded using a digital audio-recorder connected directly to the telephone.

The consultation groups with pwMS were held in local accessible venues. As fatigue is a major issue for many people with MS, we ensured that the groups lasted no longer than 90 min and included regular breaks, refreshments, and lunch. It was emphasized that participants could take a break or withdraw from the group at any time. To minimize burden, written consent was obtained on the day of the consultation groups. Following informed written consent, participants answered a brief self-report questionnaire (demographic information, MS-related characteristics, and familiarity with technology).

Interviews and consultation groups were audio-recorded and transcribed verbatim. Outcomes of the consultation were discussed at a roundtable meeting that included representatives from the consultation team, the MS Society, HCPs, and pwMS.

### Analysis

Quantitative questionnaire data were analyzed and summarized using descriptive statistics.

A generic qualitative approach to thematic analysis was used [13] with inter-researcher interpretation. Following familiarization with the transcripts, a member of the team charted themes in a matrix. Subsequently, 2 other team members familiarized themselves with the transcripts and the matrix of initial themes. They developed an agreed coding scheme using an analytical framework that combined a priori issues from the original topic guide and emerging themes [14].

## Results

### Overview

In total, telephone interviews with 8 HCPs (6 occupational therapists and 2 physiotherapists) took place, 4 of whom were identified via the project lead’s networks and 4 from an MS Society database. All HCPs were willing to be audio-recorded. All had been working as HCPs for 10 years or more and had delivered FACETS 72 times in total (mean 9 [SD 3.6] times, range 4 to 15). Four were based in the community, 3 worked in hospitals, and 1 worked in an inpatient setting. All had attended the FACETS facilitator training provided by the MS Society with the exception of one HCP who had been trained by a FACETS-trained colleague. Interview durations ranged from 36 to 81 min (mean 61 [SD 16.6] min).

All pwMS were willing to be audio-recorded. A total of 3 consultation groups were held with pwMS between May and September 2017 (London, n=4; Bristol, n=7; and Liverpool, n=4) with similar numbers of males and females participating. Most participants had attended FACETS within the past year; 3 had attended 2 to 3 years ago (based on their self-report). The mean age (SD) of the sample was 53 (12) years and time since diagnosis ranged from 1-5 years to >20 years. All types of MS were represented (Table 2).

A total of 15 people attended the roundtable meeting that was held on June 19, 2017 in an accessible building in central London. Attendees included representatives from Bournemouth University (psychology, qualitative research, and human computer interaction; n=4); the MS Society (information and support, digital, innovation, and self-management; n=6); clinical practice (occupational therapy and physiotherapy; n=3); and pwMS (n=2).

### Qualitative Themes

A total of 7 themes were identified (Table 3 and Multimedia Appendices 1-7).

### FACETS: Delivery

#### Key Aspects

A total of 7 key aspects of FACETS were identified (Table 4). The group aspect was considered central to the success of the program. It was seen to provide an environment for peer learning and sharing in which empathy, mutual support, and sometimes plain speaking could be highly beneficial. Some pwMS described how they continued to be in contact with others from their FACETS group.

FACETS integrates elements from cognitive behavioral, social cognitive, and energy effectiveness theories and principles. It aims to help pwMS to (1) understand more about and normalize MS fatigue; (2) learn how to use available energy more effectively; and (3) learn helpful ways of thinking about fatigue [7]. In the program, although there is a gradual transition from a practical to a more psychological orientation, the cognitive behavioral (CB) elements (thoughts, behaviors, emotions, and physical aspects) are introduced early. This means participants can explore these inter-related elements before the CB model is formally introduced during the fourth session. The CB aspects were considered crucial by HCPs in supporting changes in ways of thinking and lifestyle that often go beyond fatigue management. Other key aspects highlighted included provision of information, the relaxation training and practice, group activities, and the homework tasks (which translate into everyday life).

#### Positive Aspects and Changing Perspectives

HCPs highlighted that the program structure allows for personal reflections, discussion with trusted others in similar positions, and the opportunity to make behavioral and attitudinal changes with long-term impact. PwMS liked the fact that FACETS offers a positive approach to making lifestyle changes (in comparison with other groups some had attended) and opportunities to consider different ways of thinking that might pave the way for such changes.

**Table 2:** Self-reported descriptives for consultation group participants (n=15).

Variable		Descriptive statistics
**Sex, n (%)**		
	Male	8 (53)
	Female	7 (47)
Age (years), mean (SD) range		53 (12.0) 27-76^a^
**Type of MS^b^, n (%)**		
	Relapsing remitting	3 (20)
	Secondary progressive	3 (20)
	Primary progressive	5 (33)
	Don’t know	4 (27)
Adapted Patient Determined Disease Steps Scale (APDDS), mean (SD) range		4.13 (1.67) 1-6.5^c^
**Time since diagnosis (years), n (%)**		
	1-5	5 (33)
	6-10	3 (20)
	11-15	3 (20)
	16-20	1 (7)
	>20	3 (20)
**Employment status, n (%)**		
	Self-employed	2 (13)
	Unable to work	4 (27)
	Looking after house and family	4 (27)
	Retired	2 (13)
	Working full-time	1 (7)
	Working part-time	2 (13)
**Using phone for, n (%)**		
	Calling	14 (93)
	Internet browsing	7 (47)
	Watching videos	2 (13)
	Playing games	3 (20)
	Texting	12 (80)
	Calendar	6 (40)
	Reading news	2 (13)
	Social networking	6 (40)
	Emailing	9 (60)
	Listening to music	4 (27)
	Diary	6 (40)
**Use of apps, n (%)**		
	Never	2 (13)
	Use a few	10 (67)
	Use a lot	3 (20)

^a^1 case missing

^b^MS: multiple sclerosis.

^c^Possible scores on the APDDS scale range from 0-10 corresponding to 11 ordinal levels of functioning. However, one participant gave a rating of 6.5 indicating they perceived their functioning to fall between 6 and 7 on the scale. Similarly, another participant gave a rating of 4.5.

**Table 3:** Key themes identified.

Theme	Description	Multimedia Appendix
FACETS: Delivery	Comments relevant to the delivery of the FACETS program	1
FACETS: Web-based delivery	Comments relevant to the Web-based delivery of the FACETS program	2
cFACETS: Design	Comments relevant to the design of cFACETS	3
cFACETS: Group	Comments relevant to the group dynamics of cFACETS	4
cFACETS: Engagement	Comments relevant to the engagement of users with cFACETS	5
cFACETS: Interactivity	Comments relevant to the interactivity of cFACETS	6
cFACETS: HCP^a^ Relationships	Comments relevant to the relationships HCPs might have with cFACETS	7

^a^HCP: health care professional.

**Table 4:** Key aspects of FACETS identified by people with multiple sclerosis (pwMS) and health care professionals (HCPs).

Key aspects described	Example response
Group delivery format	“And somehow, somebody or something, some way to be able to troubleshoot problems. ‘Cause often that’s what the group does to each other. They help each other out. They find solutions. Sometimes you can just sit back and leave them to it and they, they do actually help each other. Which sometimes you can see, the penny drops for one person because somebody else has said it.” [HCP 1]
Group tasks and homework - how they translate into everyday life	“And the third thing is the tasks translating into real life. The tasks you’re asking people to do at home. So I think that’s one of the key parts.” [HCP 1]
Cognitive behavioral model	“I think the cognitive behavioural therapy aspect of FACETS is really important. ‘Cause that seems to be quite a big barrier I think, in terms of how people take the practical advice going forward, is in terms of how they then view fatigue management and fatigue itself. And often, again, it’s the interaction with people that helps them realise that. And then obviously, like the practical tips and hints from other people as well, so, kinda hearing what other people have tried.” [HCP 2]
Relaxation	“Another really core bit is the relaxation. Whether you can do that with a voice online, because the relaxation techniques become very important to a lot of them because they learn how they can take a quick 5 or 10 minutes while still seeming to be active at their desks. Things like that, you know when people start off by saying, “well I work. I’ve got no way I could possibly leave my desk” or “there’s nowhere to have a rest”, or “we don’t take lunch breaks”, and all those things. Getting them to re-evaluate that and start to take some breaks is one thing. But also, a quick deep breathing session when they practise, they can do it and pretty much look as if they’re still working.” [HCP 1]
Addressing thought barriers as well as providing practical management strategies	“Yeah, I think sometimes patients will challenge each other if they’re having some very unhelpful thoughts. For example, we’ve had somebody in the group who was once talking very much about doing a lot for her son and other patients were, ‘Well, he’s an adult. Why are you doing all of this for him?’ So I think sometimes they can take it better from people who are also patients, rather than from a professional as well. So obviously we can facilitate that conversation as well." [HCP 6]
Contact with a skilled and knowledgeable therapist	“I did think the group was good because you got 6 hours, no 12 hours, with an HCP who is an expert in the area who wants to help you as well and so you felt that. Just the advice, the perspective. I was kind of surprised. I suppose with my particular diagnosis there’s nothing else for me other than advice and guidance about how to just improve your health or deal with the condition. Do you know what I mean? It is so important and it was good that the recognition is there and courses have been developed.” [Participant 2–Consultation Group (CG) 3]
Length of program and the way each week builds on previous content (giving people time to reflect)	“I think a lot of it is common sense and we all know it. But it actually makes you realise that it can be addressed or dealt with maybe in a slightly different way, which you don’t get to stop and think of before. I think that’s another thing, you get time to just stop and discuss other things that you just deal with on a daily basis, but don’t necessarily do it in the best way really. Most effective.” [Participant 3—CG2]

### FACETS: Web-based Delivery

#### Pros and Cons of a Web-based Delivery Model of FACETS

HCPs and pwMS described a variety of pros and cons. These suggest that although a Web-based version would provide a desirable solution offering many benefits, it would complement rather than replace a F2F version (Textboxes 1 and 2).

**Pros of a Web-based delivery model of FACETS.**

**Pros**

- Potentially more cost-effective—would require less professional time than running face-to-face version. (health care professional [HCPs])
- Reduction of the logistical issues around delivering a physical course in a specific location each week. (HCPs)
- Immediately available to everybody—globally extending the reach to people who otherwise might not be able to access it. (HCPs, people with multiple sclerosis [pwMS])
- Addresses the waiting list issue for current course attendance in some areas. (HCPs, pwMS)
- More convenient method of delivery where pace and preferred time of learning—which might be affected by MS symptoms—could be personalized to individual needs and returned to many times if things were unclear. (HCPs, pwMS)
- Could act as a refresher or resource for those who had attended the F2F program. (HCPs, pwMS)
- Those used to interacting in online environments might prefer the online format as might pwMS who do not like the group aspect of delivery. (HCPs, pwMS)
- A Web-based delivery format could help to make some of the content more engaging by employing a wider range of audio and visual stimuli. (HCPs, pwMS)

**Cons of a Web-based delivery model of FACETS.**

**Cons**

- Loss of F2F group aspect of FACETS. (health care professional [HCPs], people with multiple sclerosis [pwMS])
- Hosting a group in an online forum is different from one in which you are meeting others in person—where the group atmosphere with the facilitator is not easily replicated and is less personal. (HCPs, pwMS)
- A Web-based solution is not as responsive as a facilitator who is able to give tailored support when needed and can also proactively head off any misconceptions held by participants or medically concerning comments. (HCPs, pwMS)
- An online participant might need to be quite confident with IT to use a Web-based solution which has some implications for the target group. (HCPs, pwMS)
- There is a reliance on an individual to be motivated to engage and take on board information—which is easier to monitor and manage in a F2F environment. (HCPs)
- Getting buy-in for a Web-based solution to be used and the associated training and support costs for facilitating in a Web-based environment (whether via audio, video, short message service, or email) could be challenging. Providing telephone support has cost and time implications. (HCPs, pwMS)
- Would lack the responsiveness of an experienced facilitator to deal with individual comments and situations as they arise. (HCPs, pwMS)

### cFACETS: Design

#### Audience Demographic

It was noted by HCPs that the age range of pwMS is broad and there will be differing attitudes to, levels of experience with, and acceptance of technology (such as what individuals choose to share online) as well as differing levels of patient activation [15]. Personal circumstances, attitudes to groups, stage of personal journey with MS, and ability to attend F2F sessions will all impact on preferences for a digital option:

Everyone’s different.

Participant 4

Everyone’s different so you give them the option.

Participant 3—CG3

The fluctuating nature of MS and MS symptoms make it particularly important to ensure that Web-based materials are easy to access and interact with. HCPs suggested that information about a person’s MS and other aspects could be requested at the beginning of cFACETS and content could be individually tailored. This concept aligns with the MS Society’s digital strategy in terms of patient activation and personalized self-management [15-17]. However, it was also noted by HCPs that mixed F2F groups seem to work well.

#### Timing and Pacing Considerations

Pacing considerations were highlighted by both HCPs and pwMS. Delivering content over a minimum of 6 weeks allows for adequate breaks between sessions, provides opportunities for reflection on the topics and homework elements, and increases the likelihood of behavior change. Suggestions also included the prompting of taking breaks within cFACETS.

#### Look or Structural Considerations

There was a general consensus that the linear structure of FACETS should be kept to ensure materials are not completed out-of-step or rushed through:

I think the order that it was delivered, because one thing, you started off as you said, looking at fatigue and types of fatigue and that led on to SMART targets which led on to the cognitive behaviour therapy, so I think it flowed quite nicely from one thing to the next so I think the order is right really. I don’t think you could do it in any other order to get out of it what we got out of it.

Participant 3—CG3

#### Formatting Considerations

When porting printed materials to a digital environment, both HCPs and pwMS emphasized the importance of ensuring they are structured into small, easily digestible chunks [18]. HCPs suggested including a mix of formats to suit different learning styles and providing summaries at the end of sections to enhance retention and consolidation. PwMS felt that an option to print homework materials should be provided in any digital solution. HCPs noted the importance of considering the intended end user of cFACETS in terms of design aspects, for example, using drop-down menus rather than requiring long segments of text to be inputted; avoiding the need for precise movements that may not be possible for those with tremor; and providing audio or video materials to complement text to reduce fatigue and concentration requirements.

#### Relevance for Important Others

Providing access to program content to others (eg, relatives, friends, carers, work colleagues, and employers) could provide greater awareness about MS fatigue and its impact. Several pwMS noted that with FACETS, this had been beneficial and they were keen to see this aspect preserved or extended:

You know because it is not like a normal fatigue. I just literally crash out but they used to get on my back and say you should just go to bed if you are tired. Trying to explain to them it is not tiredness as such. Luckily having all the info to hand I have been able to sort of say, “look this is what happens - it’s the MS, it’s not me being tired, it’s the MS” which they now understand but I think having the online, maybe you know, there could be some opportunity for your partner if they want to access some of it as well, you know, so that they understand you; know what is happening to you.

Participant 3—CG3

#### Phased Implementation Approach

At the roundtable meeting, the MS Society proposed a minimum viable Web-based version as the best starting point for a digital solution to enable core elements of content to be introduced in a timely manner. However, it was recognized by pwMS and HCPs that support elements would need to be considered to bring about and sustain long-term behavior change:

People, you know, behaviour change takes time for a reason and people need time to reflect and you can’t just whip through it, like you could do for some other online courses.

HCP 3

Yes I mean. I don’t feel that just putting slides or a presentation on a website for someone to go through will be sufficient.

Participant 6—Roundtable

Because there is a difference isn’t there between finding out about fatigue and whether one of the aims of the [online] course is behaviour change and those are two very different things aren’t they?

P1–Roundtable

### FACETS: Group

#### Online Group Ideas and Size

Capturing the group dynamics of FACETS raised challenges. One option would be to replicate the small closed groups of FACETS but in an online format. If groups were regionally organized, members could break out as a physical group to support each other following the program (which often occurs with connections formed by pwMS during FACETS).

Yeah, I think that would be really beneficial. ’Cause then you effectively have a group of people like you had here, like 8 in a room that say at the end of it, “you know what, let’s all meet up, go for a drink!”

Participant 2—CG1

#### Telephone Support/Ask the Expert

Providing telephone support during cFACETS or holding *Ask the Expert* sessions were deemed good ideas (albeit with logistical and cost implications) by both pwMS and HCPs. Making use of telephone support by a person unknown to users was not seen to be an issue by pwMS and examples were provided of obtaining support from the MS Society helpline. Web-based support was preferred to telephone support by HCPs (influenced perhaps by the perceived extra cost of providing this option). An advantage of *Ask the Expert* was that those unable to log-in for the live session could access a recording or transcript at a later date. Concerns raised included whether these support mechanisms would primarily tend to engage those who would already be calling helplines; whether they would be sufficient to achieve behavior and lifestyle changes; whether the group aspect would be lost; whether there would be adequate capacity to answer all the questions; and that *Ask the Expert* places the onus back on the HCP to have all the answers as the *expert*.

Given that regular weekly support sessions would likely be unfeasible, an introductory session was suggested as a means of building trust and promoting enthusiasm for asking questions in subsequent sessions and consolidating the key program concepts. Similarly, a closing session could be used to consider the way forward at the end. A computerized expert was proposed as a possible innovation. However, some pwMS viewed this approach as depersonalizing and potentially lacking credibility.

#### Forums

Forums were discussed as ways to enable peer discussion and sharing of experiences. HCPs had some concerns about a static forum (questions posted and responded to later by others) as it would mean that, unlike in FACETS, misinformation and problematic suggestions could not be dealt with immediately. Static forums would require continuous scanning and moderation of content (which is challenging and time consuming).

I think the only worry is, occasionally when that happens, you get, kind of, un-evidence based ideas. Like, a couple of times in our groups we’ve had people talk about or really pushing really extreme diets. Or, somebody that was really pushing oxygen therapy. And, we were able to say within that, what the evidence base says about that. So I don’t know how you quite monitor that in those forums.

HCP 4

However, most pwMS reported feeling comfortable with using forums, and there were no strong preferences in terms of a closed (restricted to cFACETS users) versus an open (available to others with MS) format.

#### Group Aspect/Webinar

Capturing the F2F facilitated group environment online was a key challenge that was identified. The use of webinars was proposed by HCPs and pwMS as an interactive way of complementing other online materials (and could either be viewed live or later). Getting a number of participants to *meet up* regularly at the same time each week was acknowledged to be more difficult to achieve online than F2F. From a logistical point of view, it could be challenging to ring-fence each separate cohort progressing through cFACETS to maintain the closed group dynamic:

There’s something different that happens when you’re physically in the room with someone. Even seeing their face online is not the same.

Participant 5—CG1

Other challenges concerned the format of the technology being used and whether sound and vision would be possible for everyone in the group or just for the facilitator. Discussions also included whether live or prerecorded sessions made available later could be used as a *catch-up* resource for those unable to attend specific sessions.

#### Trust and Safeguarding

One of the most positive aspects of FACETS described by pwMS was how meeting up regularly with the same group helped to build an element of trust that allowed close relationships to be formed. This enabled discussions about topics that might not otherwise have been discussed:

It’s a safe place. When you’re all together and expressing yourself, it’s a safe place.

Participant 7—CG1

#### Booster Sessions

Booster sessions could be a helpful complement to FACETS to enable a facilitated review of progress and barriers encountered following the program [10]. These obtained favorable feedback as a means of providing opportunities for pwMS to revisit problematic issues, discuss new challenges, and refresh key principles.

### cFACETS: Engagement

#### Keeping People Engaged or Adhering to It

It was suggested that some challenges to maintaining engagement could be addressed by ensuring that content is relevant for and useful to the user:

It’s what you get out of it, isn’t it? If you think, “I’m benefitting from this” then you’ll want to do it. If you think “it’s a waste of time” then you’re not going to bother.

Participant 1—CG2

Interruption levels and distractions may be higher in an online environment than in a F2F group. When learning about relaxation techniques, some pwMS felt that without the F2F guidance from facilitators, the Web-based content would not be as engaging to view or easy to understand.

Feedback from pwMS suggested that they found the FACETS materials engaging—one of the reasons they continued to attend the program—and sometimes returning to the materials after the program had ended elicited changes in behavior or lifestyle adjustments. However, it was noted that digitization of the materials would require reformatting; for example, providing information in brief chunks and providing feedback following completion of sections and homework elements (which could then be used as a trigger to unlock new content).

It was acknowledged by pwMS and HCPs that not everyone would have the intrinsic motivation or self-efficacy to complete a self-guided Web-based version of the program. Being part of a group makes people feel a certain commitment to attend each week. It was felt that the key motivators of the FACETS program were the benefits experienced from attending the program and a sense of accomplishment from completing it.

#### Keeping in Touch/Reminders

PwMS generally thought that reminder and notification messages would be helpful in a Web-based version so long as there was choice over their format, configuration, and frequency. Reminders could prompt users to return to the materials at specific times, provide encouragement, and could also be used for personalized, responsive messages.

#### Homework

Homework is an important element of FACETS—enabling members of the group to try out aspects covered during sessions:

The more you put into your homework, the more you’ll get out of the course as a whole. And over time, there’s always the odd one that hasn’t done stuff, but by the end of the group, even if people haven’t written things down, they’ve been thinking about it during the week ’cause they know there isn’t the pressure to write it down. And I don’t know how you would do that online.

HCP 3

Both pwMS and HCPs felt the homework tasks would transfer well to a mobile device and that this could also provide opportunities for completion reminders and symptom monitoring and management.

#### Introducing the Cognitive Behavioral Model

HCPs felt that the gradual introduction of the cognitive behavioral model (CBM) in the FACETS program works well and noted it could have been too much to take on board had it been formally introduced in Session 1:

I think if it had been in the first session, I don’t think I’d have liked that bit because it would have been just too much to take on board.

Participant 2—CG3

By the time they actually bring up the model, the person is actually familiar with all the terminology in that they’ve heard it in every week beforehand. So they've actually been talking about it the whole time, but they haven’t, it hasn’t been called that until, I think it is the 5^th^ session. So, when it finally does come up, people are really open to it because they've heard it, they've worked with it, they understand it, it makes sense to them.

HCP 4

In a Web-based delivery format, the CBM could be presented via clickable content alongside video or audio content from HCPs and pwMS to provide further explanations and real-life examples. It was suggested that these sessions may require support from an HCP and a live element of peer interaction.

#### Rewards/Gamification/Goal Setting

Various forms of rewards and gamification were suggested such as obtaining a certificate or trophies; adding elements to a virtual interactive scene or pieces to a virtual jigsaw puzzle; and giving advice to an animated character. HCPs and pwMS held mixed views about gamification with many feeling that extrinsic motivators were not necessary:

I think the reward should be from having benefitted. I don’t necessarily think that professionals should be rewarding people ’cause that kind of puts, feels almost a bit like a parent-child kind of relationship really.

HCP 6

One HCP suggested that it would be helpful if users receive some form of acknowledgment or feedback about the goals they set in any Web-based version:

If there was some way that they could continue to engage with the process and tick off when they feel they’ve achieved a goal or something, that would be quite nice.

HCP 2

PwMS noted that, in the F2F FACETS groups, attendees sometimes require additional explanations and support from the facilitators for the specific, measurable, achievable, and realistic with time for review (SMART) goal setting task in Session 3. They suggested it would be helpful to provide users with a variety of interactive examples of SMART goals in a Web-based version.

#### Progress Bar/Dashboard

Suggestions for ways to highlight progress included using tracking to document accessed sections and displaying progress on a central dashboard. Occasional user prompts and notifications could be incorporated to acknowledge progress and section completion:

It is useful to have that, how far you’ve gone.

Participant7—CG1

You might have people feeling that they can’t proceed any further if they haven’t got everything ticked...

Participant6

### cFACETS: Interactivity

#### Flipcharts

Within FACETS, flipcharts are used frequently. HCPs noted they help to keep things *fluid* and maintain momentum. Flipcharts were seen to be an important means of adding variety to sessions, highlighting links between content and identifying patterns in behaviors and thinking, promoting focused discussions, and encouraging groups who may be quieter.

One HCP noted that although using real examples of flipchart content could be engaging, care would be needed to avoid this seeming scripted or formulaic. Forums were seen as possible alternatives to flipcharts or could be used in combination with flipcharts:

So I guess in an online bit, the discussion becomes the flipchart. Because, it will be where they write and see what other people are saying.

HCP 2

#### Technical Interactivity

The use of video and audio components within cFACETS, accessible on a number of different devices, was positively received by pwMS and HCPs. It was suggested that particular elements of FACETS might benefit from having different approaches in terms of the presenter—such as having HCPs and pwMS talking to the camera about a topic or, alternatively, a group being filmed in particular situations—for example, discussing a certain issue or feeding back about a homework task:

I’m wondering if there’d be any potential to have a video of a group speaking about different things...the way [XX] has just spoken about...that’s very powerful.

Participant 2—CG3

Interactive tasks (including quizzes and polls) were also suggested by both HCPs and pwMS as engaging ways to communicate content and give the user a sense of *ownership*.

So I think with the tasks, just having more tasks, more click buttons, more things that they have to fill in so they’re actually getting a bit more ownership of it.

HCP 1

#### Real or Virtual

There was much discussion surrounding the use of real people versus virtual characters. The consensus of pwMS tended toward using real HCPs or pwMS to convey key points, rather than using avatars or cartoons:

I won’t trust them. I won’t trust them ’cause I don’t know them. They’re not real, so. Personally, I don’t respond well to avatars.

Participant 5—CG1

It was noted that having FACETS delivered by HCPs with a mix of professional backgrounds provides complementary perspectives and that pwMS could also be involved in delivering a Web-based version (or the choice of facilitator could be up to the user). It was considered important to include HCP support so that incorrect or problematic suggestions could be addressed.

Animations were considered useful but some pwMS felt they can feel patronizing and risk trivializing the subject matter. VideoScribe [19] was suggested as one example of a visual tool that could have a variety of possible uses (storytelling, conveying key concepts, and virtual flipcharts).

### cFACETS: Health Care Professional Relationships

#### Health Care Professional Involvement

Reflecting on FACETS delivery, HCPs felt that their support would continue to be necessary to help achieve long-term behavior and lifestyle changes. Without some form of additional support and facilitation, there were concerns that the shifts and movements in people’s thinking, integral to FACETS’ success, might be less likely to occur. Several HCPs advised they could, in principle, be involved in an *Ask the Expert* component so long as there were prescheduled dates and time slots organized well in advance, and the role was shared among several HCPs.

#### Supporting Health Care Workers/Complementing Care

How cFACETS could complement existing care was also considered. It was felt by HCPs that any Web-based version should not replace the F2F programs being delivered, as the F2F format works well. However, giving pwMS the option of attending a group session or accessing a Web-based version could increase reach (though technical issues, such as low internet speeds in rural areas, would need consideration).

## Discussion

### Principal Findings

The consultation feedback highlighted the positive aspects of FACETS that have made it a successful program to date—including the group dynamics, CB approach, and sequential delivery model. Conclusions from the roundtable meeting were that a minimum viable Web-based version was the best starting point as it would allow for a digital solution to be implemented that could be added to over time. As potential app-based solutions were outside of the scope for digitizing FACETS in a minimum viable form, another recommendation was that a separate project should look to create a free, stand-alone digital toolkit focusing on the FACETS homework elements and possibilities for real-time symptom monitoring and management.

In their systematic review of MS apps present in US app stores [20], Giunti et al found that, compared with other long term conditions such as cancer and diabetes [21-23], there were relatively few apps available (n=25). Van Kessel et al [24] similarly noted that there is currently limited access to F2F cognitive behavioral therapy (CBT)–based interventions. Three authors of the current article (PK, ST, PT) have collaborated with van Kessel and others to create a self-guided CBT fatigue management app—*MSEnergise*—for iOS [25]. Usability and field testing are currently underway.

A complementary mobile solution enabling the FACETS homework elements to be made interactive and portable aligns closely with recommendations from the MS Society *Data and Technology* Report [16] and Action Plan [17] (specifically the areas of having more control over care and accessible and coordinated care). It would also help to meet the aims of the MS Society research strategy concerning self-management and implementation [26] and help address the third (fatigue) and fourth (self-management) James Lind Alliance research priorities for MS [3].

Giunti et al have called for much greater involvement of pwMS and HCPs before digital solutions are implemented [27,28]. In a number of health care implementations offered to date, user requirements, existing patterns of use, and HCP reflections have not been considered before or during the development of a solution [29,30]. It is imperative that consideration is given to the requirements of pwMS throughout the development, prototyping, and implementation of any digital solutions [16,17,31]. Feedback from this consultation has highlighted the opportunities and challenges of designing for an online audience of pwMS in terms of user requirements, design elements, and structural and pacing considerations.

A paced delivery format would mean that group members could work through the cFACETS program as a closed cohort. Although such a model could promote the idea of a group identity, it might undermine the flexibility and potential strengths of Web-based delivery. It was deemed key to design a Web-based version that could be accessed by, and have relevant content for, family members, friends, and work colleagues in addition to pwMS. Capturing the group element of FACETS in any digital solution was seen as a priority while at the same time not compromising on aspects of trust or safeguarding. However, it might not be possible to capture this aspect of the program fully online. Safeguarding issues were raised, including the importance of ensuring procedures were in place within any online group environment should significant disclosures be made or fake profiles be exposed [32].

Research is currently underway with French colleagues to develop and test the suggested concept of booster sessions [33]. Web-based booster sessions could offer cost and time efficiencies and be designed as a menu of key concepts and tools, incorporating a number of formats such as *Ask the Expert*, webinars, and chat forums.

Maintaining engagement with a Web-based program presents considerable challenges [34-38]. Responses highlighted the importance of considering the most effective ways of maintaining engagement. First, how configurable reminders, gamified elements, rewards and the use of progress bars and dashboard layouts might encourage engagement and adherence [28] and limit dropout [35]. For example, providing help buttons, linking goals via a mobile phone and providing real-life examples from pwMS could be ways to address engagement with SMART goal setting and completion. Second, how offering scheduled booster sessions digitally once the Web-based program has ended might improve long-term engagement [33]. Additionally, suggestions were made regarding how best to make use of the program’s homework elements to ensure they are accessed, completed, and reflected upon and how best to exploit the strengths of a digital format when introducing complex elements such as the CBM [7,10]. In FACETS, those who have not completed the homework still obtain benefits from *check-in* discussions at the start of each session. On the Web this could take the form of videos of people discussing the homework or chat forums that focus on the homework. Diaries and completed homework tasks could feature in a dashboard—but design and functionality aspects would require careful consideration to ensure tracking features are not overbearing.

Although interventions using Web-based approaches have shown promise within health care and in the support of pwMS [39,40], one recommendation from Beatty et al’s [41] study of an intervention for cancer-related distress was that future Web-based programs should be multi-platform in nature (so interventions could be used across the full range of smart devices and computers, enabling greater access). Findings from a recent RCT of a German CBT self-guided interactive Web-based intervention for MS fatigue were promising though dropout was relatively high in the intervention group (26% at postintervention follow-up) [42]. This again highlights the importance of considering issues of adherence and engagement when designing and developing digital solutions.

Interactive approaches were seen to be highly important to enhance and maintain engagement and enable personalization [16,17]. Examples were given from FutureLearn [43], where a number of short videos are located on different pages, with transcriptions available below. Suggestions were made regarding how session elements, such as flipcharts, could be made interactive in a Web-based delivery format. Metaphors and analogies presented visually could help to convey aspects of invisible symptoms [44]. Other suggested options for improving engagement included using video and audio elements to either replace or complement different program sections and using appropriately pitched interactive characters—animated therapists have been successfully used in other interventions [45]—and interactive quizzes and polls.

Participants also suggested how a Web-based solution could potentially link with HCPs in terms of delivery of and complementing existing care, alongside the continuation of the successful F2F program. There are potential benefits from enabling clinical teams to interface with an online solution—such as possibilities for remote monitoring and support. HCP involvement in cFACETS would vary depending upon the types of solutions suggested and their resource and workload implications. An online solution could be offered with optional support to accommodate differing preferences, needs and levels of patient activation [16,17]. If technology allowed, an enhanced support version could include an option for members of an individual’s clinical team to check-in and see how they are doing and for progress alerts to be sent. This could allow pwMS to send questions to members of their own MS team or be able to view details of their MS team via a dashboard when invited to take part. It could also provide an information resource about fatigue for HCPs and other professionals supporting pwMS.

Providing a digitized version of FACETS opens up opportunities for different evaluation methods and ways to enhance and measure reach and impact. Thought should also be given to a wider evaluation of the current FACETS program than previously undertaken [12], as anecdotal evidence from this consultation suggested that the cumulative impact is under-reported.

Digitizing FACETS also presents challenges in terms of successfully replicating group dynamics—a key program component—and mirroring the pacing of the 6-session format where concepts and materials are introduced in a structured and staged manner allowing time for familiarization, reflection, and practice. Findings from the consultation suggested that consideration be given to the inclusion of a human support aspect to maintain adherence and increase the likelihood of behavior change [46-48].

Even acknowledging these challenges, the potential of a digital environment should not be overlooked. Relative to other long-term conditions, MS is currently under-served by health technology. New technology enhancements offer opportunities for personalized electronic health solutions relevant to pwMS and the management of fatigue [16,17] such as voice-activated speakers [49] and the ability to collect live biometric data by using plug-in oximeters and wearable monitoring devices (although accuracy still needs improving) [50]. There is also the possibility of exploring future integration with existing data streams, such as linking data recorded about FACETS attendance and from the homework tasks (such as goal setting and future plans) to a national MS register [51].

### Limitations

The consultation discussions did not fully cover how pwMS were currently utilizing technology and the issues they had encountered. These limitations are being addressed via consultation groups with pwMS that will provide additional information for the initial design requirements of the digital toolkit. A further limitation was that no MS nurses were interviewed. Attempts to recruit MS nurses proved unsuccessful, but the majority of HCPs who have attended the FACETS training and delivered the program in clinical practice are occupational therapists. As the consultation did not include pwMS who have not attended FACETS, those who volunteered to participate in the consultation were likely to have relatively high levels of patient activation and hold a positive view of the program. This limitation can be addressed during the development of cFACETS and the digital toolkit by obtaining insights from a wider group of pwMS (including those who have not attended FACETS), using an online study and theory-led approach such as that piloted by Apolinario et al [52].

### Conclusions

A minimum viable Web-based version of cFACETS was considered the best starting point, enabling a phased solution that could be added to over time. It was also suggested that creating a free, stand-alone digital toolkit focusing on the homework elements of FACETS could add value to cFACETS and fill a missing gap in mobile health for pwMS. Funding for an initial version of the digital toolkit has been obtained. The first version is in development with close involvement from pwMS during the design and build phases [53]. Meaningful involvement of pwMS is essential in all stages of development, prototyping, and implementation to achieve a user-centered solution.

## Appendix

Multimedia Appendix 1 FACETS Delivery Comments.

Multimedia Appendix 2 FACETS Web-based Delivery Comments.

Multimedia Appendix 3 cFACETS Design Comments.

Multimedia Appendix 4 cFACETS Group Comments.

Multimedia Appendix 5 cFACETS Engagement Comments.

Multimedia Appendix 6 cFACETS Interactivity Comments.

Multimedia Appendix 7 cFACETS HCP Relationships Comments.

## Abbreviations

CB: Cognitive behavioral
CBM: Cognitive behavioral model
CBT: Cognitive behavioral therapy
cFACETS: Digitized version of FACETS program
CG: consultation group
FACETS: Fatigue: Applying Cognitive behavioural and Energy effectiveness Techniques to lifeStyle
F2F: face-to-face
HCP: health care professional
MS: multiple sclerosis
pwMS: people with multiple sclerosis
RCT: randomized controlled trial
SES: standardized effect size
SMART goals: Goals which are specific, measurable, achievable, and realistic with time for review
