# Supplementary material for: Digitizing a Face-to-Face Group Fatigue Management Program: Exploring the Views of People With Multiple Sclerosis and Health Care Professionals Via Consultation Groups and Interviews
Source: JMIR Form Res. 2019 May 22;3(2):e10951. doi: 10.2196/10951 (PMC6549474; doi:10.2196/10951)
Supplement: Multimedia Appendix 1 [file formative_v3i2e10951_app1.docx]

### Appendix 1: FACETS Delivery Comments

Comments relevant to the delivery of the FACETS program

| **Category** | **Example responses** |
| --- | --- |
| **Key Aspects of FACETS: Communication / group dynamic** | *And one of the things that FACETS does, that I’ve found in sessions when delivering them, is that the people challenge each other. And that’s completely, completely different to when a healthcare professional might say, “Oh is there another way of thinking about things?” I think the participants themselves are a little more direct. And, I think that’s one of the things that, where it really wins. And what sets it apart and what makes it very different and a very successful programme. Which I think it is, I think it’s a brilliant programme. Hence the reason you stick with it and deliver it so many times. [HCP 7]*  *And somehow, somebody or something some way be able to troubleshoot problems. ‘Cause often that’s what the group does to each other. They help each other out. They find solutions. Sometimes you can just sit back and leave them to it and they, they do actually help each other. Which sometimes you can see, the penny drops for one person because somebody else has said it. [HCP 1]*  *Yeah, I think sometimes patients will challenge each other if they’re having some very unhelpful thoughts. For example, we’ve had somebody in the group who was once talking very much about doing a lot for her son and other patients were, “Well, he’s an adult. Why are you doing all of this for him?” So I think sometimes they can take it better from people who are also patients, rather than from a professional as well. So obviously we can facilitate that conversation as well. [HCP 6]*  *But certainly if the group starts talking about something that they find is really significant, we could almost not be there sometimes. And my perspective on the whole thing, the whole FACETS thing is the more they’re talking, the better really. [HCP 4]*  *…so whatever you lay on. As we were saying, the exercises and homework and making you think about it, the 3Ds, whatever. They’re all really useful. But what’s the really useful thing is meeting the other people- [P6 – CG1]*  *…anyway I was diagnosed with MS I was going downhill ‘til I went on the FACETS course; really enjoyed it got a lot out of it. It was like as though I was able to accept it and I got permission to feel the way I did and it was nice to be in the group with people going through the same... [P4 – CG3]  I think to be honest before I went on it I think I would’ve chosen the one-to-one but after going on it I think that the group was highly beneficial. [P2 – CG3]* |
| **Key Aspects of FACETS: Cognitive behavioural model** | *I think the cognitive behavioural therapy model is a key aspect and that can be adapted and there’re lots of models already running that show you can do that. So I don’t think that’s a big barrier. [HCP 4]*  *I think the cognitive behavioural therapy aspect of FACETS is really important. ‘Cause that seems to be quite a big barrier I think, in terms of how people take the, the practical advice going forward, is in terms of how they then view fatigue management and fatigue itself. And often, again, it’s the interaction with people that helps them realise that. And then obviously, like the practical tips and hints from other people as well, so, kinda hearing what other people have tried. [HCP 2]*  *It’s really, it’s much more powerful than I thought it was going to be. I thought it would just be running a fatigue management programme and, but it’s really, you know, not for everybody, but for some people, it’s a lot of movement, shifting of ideas.* [HCP 3]  *It’s really useful to look at the thinking patterns of people and looking at the thoughts, unhelpful thoughts and how they might be challenged. ‘Cause I think lots of fatigue management programmes and information focus on the techniques and the strategies that can help. But they don’t always focus on, perhaps what the barriers might be in thinking processes as well.* [HCP 6]  *I do think the cognitive behavioural theory approach is really, really good as well. That’s what sets it apart from other educational programmes. Getting people to analyse their thinking behind the actions they take. So, the information giving and the CB theory approach is key to it.* [HCP 7] |
| **Key aspects of FACETS: Other** | *Another really core bit is the relaxation. Whether you can do that with a voice online, because the relaxation techniques become very important to a lot of them because they learn how they can take a quick 5 or 10 minutes while still seeming to be active at their desks. Things like that, you know when people start off by saying, “well I work. I’ve got no way I could possibly leave my desk” or “there’s nowhere to have a rest”, or “we don’t take lunch breaks”, and all those things. Getting them to re-evaluate that and start to take some breaks is one thing. But also, a quick deep breathing session when they practise, they can do it and pretty much look as if they’re still working. [HCP 1]*  *And the third thing is the tasks translating into real life. The tasks you’re asking people to do at home. So I think that’s one of the key parts. [HCP 1]*  *And also, they always remember the judge. Is it in session 5 or 6? ‘So would you judge your friend so harshly?’ And that really resonates with them. [HCP 7]*  *I did think the group was good because you got 6 hours, no 12 hours with an HCP who is an expert in the area who wants to help you as well and so you felt that just the advice, the perspective. I was kind of surprised. I suppose with my particular diagnosis there’s nothing else for me other than advice and guidance about how to just improve your health or deal with the condition. Do you know what I mean? It is so important and it was good that the recognition is there and courses have been developed. [P2 – CG3]*  *For me it was the coping mechanisms that we talked…. there’s different strategies really to cope when you’re tired and fatigued and I think someone mentioned about giving yourself permission to rest. [P1 – CG3]* |
| **Positive comments and Changing perspectives** | *I’ve only been to one other MS group where it was trying to deal with MS. That group was very miserable. But, the good thing about FACETS was, it’s right we’re going to get back to work, try and maybe do things slightly differently, we’re going to do it this way instead of that way. I thought that was really brilliant. [P4 – CG2]*  *I think a lot of it is common sense and we all know it. But it actually makes you realise that it can be addressed or dealt with maybe in a slightly different way, which you don’t get to stop and think of before. I think that’s another thing, you get time to just stop and discuss other things that you just deal with on a daily basis, but don’t necessarily do it in the best way really. Most effective. [P3 – CG2]*  *It’s finding out, for myself it was a case of, I know it sounds daft but I’m actually ill, it’s that realization, I knew but I am actually ill, I’m probably not going to get any better, this is as good as it’s going to get. So it’s the acceptance of that and then the acceptance of understanding why I’m ill and what issues are affecting me and accepting that as well because you know, you just think, been exerting again and carry on with life but understand this is a progression and then once you’ve come to terms with that then these are some of the strategies you can put in place for it. [P1 – CG3]*  *And people who would stop me in the corridors and would thank me and say, “thank you so much for that, that really changed my life, that programme.” [HCP 4]*  *I know that the first session we do, when they bring their carers, people really enjoy that. People with partners were like, “wow, I just didn’t realise, I thought I understood it, but I really don’t understand it.” [HCP 8]* |
| **Current evaluation of FACETS** | *I do. But I do just one question. I do the modified fatigue, MFIS. Modified Fatigue Impact Scale. So I do that pre. They bring that with them, completed. It’s sent out with their pack. You know when they’re invited. And then I do the follow-up immediately at the end of session 6. [HCP 1]*  *No I don’t actually. No. That’s the one thing actually. We’re doing FACETS at the moment, that we haven’t been taking outcome measures. Because obviously FACETS was demonstrated to be effective, we didn’t take the outcome measures, but that is something I’m considering we start at some point. [HCP 2]*  *We use the MSIS-29, is that what you want to know? The NFI, WHOQOL-BREF, and the self-efficacy scale that [Thomas et al.] developed [MS-FSE]. And then at the end we do an evaluation form, you know, ‘how was the parking, were you given enough information, what are the top three things that you learned from FACETS?’, that kind of thing. [HCP 3]*  *We, so we just keep a record of fatigue scores, before and at the end of the course and we just do like a, we have a [NHS] Trust-wide evaluation form for courses that we just get them to fill in and we just keep. [HCP 8]* |
| **Outcomes and impact important to measure** | *It would be interesting to know how, did they just get the ‘head knowledge’ or did they apply it? I don’t know how you measure that! But, wouldn’t it? ‘Cause that’s the thing about an online thing, they can read the participant manual and get all the information, but they’re not actually going to engage with it. So I don’t know how you can measure, other than maybe the homework things or the goal setting. I like the goal setting and maybe you could do mini-goals each week. And then just say whether you’ve managed to achieve or did you partly manage to achieve or... [HCP 5]  Personally, I think the measurements of fatigue and fatigue impact is fraught with difficulty ‘cause of how subjective it is. And, it’s just, and every study uses a different measure and blah, blah, blah. I actually don’t really truly trust the measures. [HCP 4]*  *I think just how it’s improved their ability to participate in everyday activities and also, quality of life as well. ‘Cause it’s not just about treating the fatigue, it’s more about how it impacts on their daily life and how they’re able to implement strategies to help improve their performance and satisfaction in daily life, areas that are important to them. [HCP 5]*  *I imagine you’d see that the benefit on a grander scale if you put them together as well. Goal recording is much harder with a big outcome measure in a trial, but I think the self-efficacy would be a really important one. [HCP 4]*  *So, that would be an interesting thing to see as well. Just a few studies have followed up long-term, so that would be useful. Probably the main thing I’d be interested in. And maybe some qualitative feedback to see what people felt they got out of it, or what they didn’t. [HCP 4]* |
