# Supplementary material for: Digitizing a Face-to-Face Group Fatigue Management Program: Exploring the Views of People With Multiple Sclerosis and Health Care Professionals Via Consultation Groups and Interviews
Source: JMIR Form Res. 2019 May 22;3(2):e10951. doi: 10.2196/10951 (PMC6549474; doi:10.2196/10951)
Supplement: Multimedia Appendix 2 [file formative_v3i2e10951_app2.docx]

### Appendix 2: FACETS Web-based Delivery Comments

Comments relevant to the Web-based delivery of the FACETS program

| **Category** | **Example responses** |
| --- | --- |
| **Pros of a Web-based delivery model of FACETS** | *Well I think the pros are that it’s accessible for some people. And that is particularly pertinent in a regional centre. I think it’s also pertinent for people that are working. And I think, some people feel comfortable using IT, rather than coming to a group. And I imagine, when it’s all costed out, that there will be some cost benefits. [HCP 3]*  *Another pro might be that people sometimes will come to FACETS and then drop out and then they never have the opportunity of going back into it again. Or, it’s much harder for them to go back into it again because they have to contact you and tell you they didn’t like it, but they’d like to come back and try it again, but do they have to try the whole thing, etc. Whereas, with the digital format, people could come in and out of it as they want to, when they are more ready to accept something or to change something. And also maybe it feels a little less confrontational because they can do it anonymously, potentially, depending on how it’s set up. [HCP 4]*  *And yeah, just suppose, most people have commented about the social part of coming on the group, being very beneficial for them. I suppose another pro is that we might be able to see more people, more frequently and it might take up less time than actually running the programme in the unit. [HCP 6]*  *No travel. No time travelling. No energy spent travelling [P4 – CG2]*  *You know the whole advantage is you are not having to sit for 2 hours you can fit it round your lifestyle.[P3 – Roundtable]*  *I think one of the benefits of online which probably would have helped me had I not gone to the group was bringing my [spouse] on board. Because that was one of the problems that I had trying to get [them] to understand….I just tend to crash out…and [they] just kept saying to me, “well why don’t you go to bed if you are tired?” [P3 – CG3]*  *…as I say it would definitely help us in the rural area because it would mean that people that couldn’t make it to the group would still be able to access the information and obviously it would cut down the therapist visits and therapists’ time, being able to do it online rather than a therapist having to go to their house to do it. [HCP 8]*  *If it was decided through the research that this kind of intervention would work with highly motivated people, then that would take them off our waiting list……. Those people that had more skills in self-management would be accessing something like this and that would free up our time to be spending more time with the people that were less able to self-manage and needed more assistance with that. [HCP3]* |
| **Cons of a Web-based delivery model of FACETS** | *I guess you have, you’re relying on somebody to, you’re relying on the individual to take it more on board. But I guess if they don’t, that’s just life…. and then you know, don’t you? You know it won’t work. [HCP 1]*  *If I was looking online I’d just want a cure, I’d want an answer, “Right how do I get rid of my fatigue?” As it happened, it was small bits of answers that have helped me, I haven’t got rid of it but small bits of answers over 6 weeks, 2 hours, have revealed to me a way that I can cope better. Whereas if I’d gone online perhaps I’d be skimming through, as you say, looking…. “where’s the answer, where’s the cure?” I’d be going to the end for say a recap, this is a recap of what we’ve done so I’d probably end up looking through the recap. Whereas you go on the course, I know you keep saying that you have to develop an online version, you go on the course you know you’re going to give 2 hours of your day up. [P1– CG3]*  *But maybe, even keeping concentration on IT might be harder for some people, than for others. The visual side of it might be a problem, in terms of having to look at something the whole time. Or if it’s auditory, some people find it really hard to maintain concentration just through verbal contact over long period. So I guess whether individual abilities and difficulties will affect which style would be better for them, or not. [HCP 4]*  *Whereas, and it’s kind of pitching it. So my different groups or different individuals will be at different levels of experience and, for example, one of my [attendees] is a journalist. So we were able to go into the fatigue, particularly in [their] job and when [they] need energy and what sort of things [they] find difficult. So that professional intervention to your specific….maybe people might miss out on. [HCP 5]*  *And I suppose it’s not quite as adaptable. So you could make it as adaptable as possible online but not, what’s the word, it’s not like, you know if someone’s really not understanding something we can give them however many examples as possible until they can understand it. I suppose it’s just the way that some people will go through and be afraid to ask or not understand it. [HCP 8]*  *Cons are losing the group dynamic, the relationships [P2 – CG2]*  *That may be helpful enough for some people, but whether people actually like coming away and out of the situation as well. We had somebody come to the group who was talking about having quite a lot of stress at home and I think maybe sometimes just getting out of the environment they’re in and having an opportunity to just focus on the programme, its content, can be helpful as well. It depends on their life at home, how many distractions, or whether they’ll be accessing this, or whether they’ll be able to focus. [HCP 6]*  *But, a lot of what happens in FACETS is challenging what people are thinking and the way they have done things. We’re assuming, doing it online like this, that the person would be able to analyse what their negative behaviour is, or their blocking behaviour, or however you want to describe it. And that they will think, “oh yes, that’s not great. If I do it this way.” Whereas when you’ve got a group, therapist, and all the rest of it, then you can then say within the group, “Well, have you thought about doing it this way?” or “Have you thought about only doing part of that?” or “What about this?” or “What about…..?” You know, you’re throwing things in all the time. Do you see what I mean?[HCP 3]* |
