# Supplementary material for: Digitizing a Face-to-Face Group Fatigue Management Program: Exploring the Views of People With Multiple Sclerosis and Health Care Professionals Via Consultation Groups and Interviews
Source: JMIR Form Res. 2019 May 22;3(2):e10951. doi: 10.2196/10951 (PMC6549474; doi:10.2196/10951)
Supplement: Multimedia Appendix 3 [file formative_v3i2e10951_app3.docx]

### Appendix 3: cFACETS Design Comments

Comments relevant to the design of cFACETS

| **Category** | **Example responses** |
| --- | --- |
| **Audience Demographic** | *Yeah, ‘cause obviously, it depends on age range. I’m old school. I’m a pen and paper person. None of this high-tech for me! But obviously, if you’re young, then you would want to be creating puzzles and things like that. And obviously, MS, you have young persons and you have geriatrics, like me! Each one to their own, isn’t it. [P1 – CG2]*  *P4: Everyone’s different P3: Everyone’s different so you give them the option [CG3]*  *I have had one experience in the group where somebody came who had progressive disease, and she actually stopped coming ‘cause she felt that she was a lot different than the rest of the people that were coming to the group, who were relapsing. So she decided that it wasn’t for her and it didn’t make her feel particularly comfortable. [HCP 2]*  *But, each group we run is slightly different because of the contribution of the group itself. And we had one group where it happened to be lots of young women and we had lots of fatigue strategies about how to manage dating. You know, it’s just, really interesting and I don’t know how you quite engineer that online. But I’m sure there’s a way around that, I’m sure there is. [HCP 4]*  *Maybe some people would find it easier to talk online than face-to-face, ‘cause things are changing, aren’t they? You know, sometimes people find it much easier, you know, younger people. [HCP3]*  *I personally think people get more out of it by having the group element. But there are clients that have refused to come along to some of the sessions because they say they’re really private and they don’t like doing groups. So, everybody’s different. As healthcare practitioners, we should be trying to offer a treatment, and this is a treatment that suits the particular needs and particular personalities of the client group that we’re seeing. [HCP 7]* |
| **Timing and pacing considerations** | *And I really think, the time is really important. So trying to make it a 2 week course say or a 3 week course. People, you know, behaviour change takes time for a reason and people need time to reflect and you can’t just whip through it, like you could do for some other online courses. [HCP 3]*  *So you must have evidence of how long people can concentrate effectively. And you might be able to do some theory and then do a little exercise they can do themselves, and then go back and do a little more theory. Or, and build in the, “OK, remember, this is about managing your fatigue, so just pause it here, and stand up, go to the loo, get yourself a drink. Open the windows, get some fresh air and then we’ll go onto the second part of it.” And again that’s building in really good fatigue management strategies, isn’t it? [HCP 7]*  *I think it should be locked sessions. ‘Cause I think what we experience, it was the right amount of information, correctly structured in the right amount of time. [P2 – CG1]*  *I don’t think everything straight away, because I think you would just be overwhelmed with…. I mean depending on how it was delivered, I think the weekly sessions were good because you had time to look at what you’d talked about and think about what you talked about and leading on to the next session. I think if you have the information all at once people tend to just skim, I mean I’m guilty of doing that anyway. You just skim through it and you wouldn’t have the same benefits as having sessions, sessional, so maybe once a week it could be downloaded rather than having everything, you know available at once. [P3 – CG3]*  *We tried shortening it. We tried doing 2 sessions in one week because we had people here [inpatient setting], and it didn’t work. In that I think you need a week to apply the message of the week. I felt they needed the week to keep focusing on, whatever they were told to focus on. Whether it was fatigue or activity, or their sleep. If you do too much, if you squeeze it into a shorter space, of time, I think you get less out of it.[HCP 5]* |
| **Look or structural considerations** | *I think it should be structured as it is, I don’t think it ought to be, I don’t know if this is what you were suggesting that you could do, one bit of it and then another bit of it out of sequence. I think it needs to be sequential, like it is. [HCP 3]*  *I think what was important for me with the FACETS course was that it was split into 6 manageable sections, focused and the way that you designed it was, at the end of one you got some sort of homework which led into the next so that was sort of helpful for me to understand it in that sort of process, so I think that would have to be replicated online.[P2 – CG3]*  *I think the order that it was delivered, because one thing, you started off as you said, looking at fatigue and types of fatigue and that led on to SMART targets which led on to the cognitive behaviour therapy, so I think it flowed quite nicely from one thing to the next so I think the order is right really. I don’t think you could do it in any other order to get out of it what we got out of it. [P3 – CG3]*  *I suppose the worry is that if you had them all open at the same time then you might get people who just plough through them all in one day or but then I suppose that’s their choice if they wanted to do that. But I suppose you could say, Session 1 will be open this week, Session 2 will be open next week, but you don’t have to do it at a certain time of day. You can do it at any point that week that you want. [HCP 8]*  *P1: Yes, access, choose yourself really. P3: Pick and choose. You don’t need to necessarily do it in the order that we did it. F: So you want all the content to be available and all at once? P3: Like a box set.  [CG2]* |
| **Formatting considerations** | *…So that’s why it’s really useful having different activities and stopping, people doing stuff in pairs, to mix it, it’s about mixing it up again, isn’t it? [HCP 3]   I don’t think you’d get a 6 weeks of 2 hours online. It would really need to be broken down. [HCP 7]  …so the option to print it out and certainly the homework sheets if you’ve got access to a printer, because it’s easier, I mean I keep mine in the kitchen so I could just pop things on it easily. Whereas if your computer, say you’ve got a desktop, say in another room or upstairs or whatever you’d have to keep popping up there and popping back down. [P3 – CG3]*  *Not using long complicated words…you know have it fairly simple you know sort of everyday language and explaining for instance cognitive behavioural therapy if you don’t know what that is; you know little explanations of phrases that are used that if someone doesn’t know what it is they can maybe click on as you say the question mark…. but having the opportunity to do so; having it highlighted so that you can actually click on if you don’t understand what it is and get a simpler explanation. That would come with the SMART goals as well if you haven’t done them before. [P2 – CG3]* |
| **Relevance for important others** | *You know because it is not like a normal fatigue. I just literally crash out but [they] used to get on my back and say, “You should just go to bed, if you are tired.” Trying to explain to [them] it is not tiredness as such. Luckily having all the info to hand I have been able to sort of say look this is what happens it’s the MS it’s not me being tired it’s the MS which [they] now understand but I think having the online maybe you know there could be some opportunity for your partner if they want to access some of it as well, you know, so that they understand you; know what is happening to you– because that has been quite a big issue for me. [P3 – CG3]*   *I mean, I know that our clients find the folders really helpful to take away and particularly for their partners to read and for their partners to be involved. So whether you could open it up as well for significant others of MS to be able to access some of the information or have their own area where they could access and support each other as well. [HCP 8]   Yes, yeah. I mean I suppose, people are motivated to support people, so if there was a forum online [for important others] or even if it was separate from the other people, at least they could get, that might be an opportunity to increase their awareness. And you know, if they’re at work and can’t obviously attend the first session, it does give that opportunity. [HCP 6]* |
| **Phased development/ implementation approach** | *P5: And for me it would be interesting to go back to what are the 3 most important needs that a minimum viable product is trying to address? And then if we start from there; from that then we can build on some of the niceties that would make a difference between being OK and being a wow factor. [Roundtable]*  *And I think what we need to be mindful of is the fact that we need to prototype cheaply and quite quickly so we can do testing. So not be too concerned about technology at this stage and then trying to fit technology further down the line. [P5 - Roundtable]*  *Once we get the minimum thing running the rest is an add-on that we will almost worry about separately. [P4 - Roundtable]*  *And all the other support that they can access, local groups, any sort of add-ons, all these other options. I think what we’re saying is probably have that first minimum viable product informing them and then move on onto the next stage. [P2 - Roundtable]*  *Because there is a difference isn’t there between finding out about fatigue and whether one of the aims of the [online] course is behaviour change and those are two very different things, aren’t they? [P1 – Roundtable]* |
