# Supplementary material for: Digitizing a Face-to-Face Group Fatigue Management Program: Exploring the Views of People With Multiple Sclerosis and Health Care Professionals Via Consultation Groups and Interviews
Source: JMIR Form Res. 2019 May 22;3(2):e10951. doi: 10.2196/10951 (PMC6549474; doi:10.2196/10951)
Supplement: Multimedia Appendix 4 [file formative_v3i2e10951_app4.docx]

### Appendix 4: cFACETS Group Comments

Comments relevant to the group dynamics of cFACETS

| **Category** | **Example responses** |
| --- | --- |
| **Online group ideas and size** | *I think a small group really ‘cause I think you‘d have that many people asking questions. [P4 – CG3]  Yeah, I think that would be really beneficial. ‘Cause then you effectively have a group of people like you had here, like 8 in a room that say at the end of it, “You know what, let’s all meet up, go for a drink.” You know what I mean? So they’re meeting…. that relationship…. in their area of people who are going through a similar thing that they could socialise with. [P2 – CG1]*  *If you have got an open group and someone is on week 6 and you’re on week 2 they’re going to be giving you different…. [P6 - Roundtable]  That’s why I think you need somebody in charge of it if you’re doing a group. You need a facilitator that you know, is going to be able to email people and say sorry you weren’t able to log in on week 2, hope everything’s ok? [HCP 3]*  *I mean obviously, groups is the best, live interaction is then the best, presenter is then the best, with people that are live and then just messaging and then just getting your information to people who are newly diagnosed that are really needing some basic information. [P6 – CG1]* |
| **Telephone support / ‘Ask the Expert’** | *[talking about MS Society contact or Helpline] As long as it was somebody that understood about MS and the impact and the effects of fatigue etc that would be ok coz I didn’t know [FACETS facilitator] that well …I have seen them a couple of times in clinic and I think I met [other FACETS facilitator] once so I didn’t really know them well but they were there; they understood, if you like. So having somebody that understands …could understand the impact and maybe talk you through things. So it wouldn’t matter as long as they understand about MS. [P3 – CG3]*  *[talking about Ask the Expert] P2: But the one thing that I’ve experienced that annoys me with that sort of thing is you tend to get generic, cut and paste back…….’cause it’s like there’s no personalisation there.*  *P5- Hopefully it would be more personalised responses.[CG1]*  *I don’t think it would be sufficient. I think it would be useful. But, I mean, I suppose if you’re really wanting people to get what a group gets out of it. I, just my own experience, I found that people needed a little more support. So even with the goal setting. They needed a bit of, very few were able to just go away and do that on their own. They needed to think about an idea and have some support of talking it through before they really crystallized it. And, without the goal setting, I think it’s hard. I think people are less likely to actually maybe implement it. [HCP 5]*  *…not necessarily with my own neurologist, but with someone, going back, if you had a launch event, someone that met that you knew to be the expert in that group. So, if you’ve met them, I would feel more inclined to ask them a question. [P5 – CG1]*  *I think ideally, it would. Because I think it gives people, certainly at the beginning or at the end of the programme, at least to build rapport and answer any immediate questions, it might be helpful. And also just at the end to see if there’s anything outstanding that maybe hasn’t been addressed by the programme. If there’s anything else that they would find useful. [HCP 6]*  *I think it would be something that would need to be trialled. I suppose the benefit of having the group programme at the moment is that the people can come and all chat together. Whereas, if it was a question and answer session with just a professional, then it kind of puts the onus back on the professional to have all the answers. And maybe it has to be more collaborative I think, working together. Whereas I think that it might be more of a question and answer session. Which is still useful, but yeah, I think it’s nice if other people can be involved in that discussion as well. [HCP 6]*  *I don’t think people want phone calls saying, “How’re you getting on with it?” really... Unless it was a planned and scheduled thing, “You’re starting the course and we meet at the beginning of it. I’m going to phone you on Friday and the middle of it” or something. Not unless they’ve agreed to it, previously. I think they might find that annoying. [HCP 5]*  *I suppose you could have like a section within, if you’re going to have like a forum, you could have like a, if you got different tabs, you could have ‘Frequently Asked Questions’, and then you could maybe have an expert answering the questions but like video rather than just like a read. “How do I do this?” and it could be an expert but it doesn’t have to be a health professional, that could be a patient going, “Well I found it was best to do it this way” or, and someone else saying, “Well I found I could do it this way.” [HCP 8]* |
| **Forum** | *I think the only worry is, occasionally when that happens, you get, kind of, un-evidence based ideas. Like, a couple of times in our groups we’ve had people talk about or really pushing really extreme diets. Or, somebody that was really pushing, oxygen therapy. And, we were able to say within that, what the evidence base says about that. So I don’t know how you quite monitor that in those forums. [HCP 4]*  *And as for the group support, I guess whether you had a forum or whether I suppose, because I’ve done online courses before where you’re almost enrolled as a group and so you can introduce yourself as a group and that group goes through the programme together. So it’s not just like a huge forum that’s open to everyone like, you know, a Facebook MS Group forum where there could be 600-700 members. [HCP 8]*  *I was just thinking about the group side of doing this online as well, 'cause that represents a challenge, doesn’t it? What could you do and how could you do that? You might be able to have discussions boards associated with the app itself or some kind of mechanism where people who have participated in the online experience can share information between themselves. I know there’s all sorts of stuff around confidentiality that they might have to tick a particular box, but there’s no doubt ways where people can do that. So many other websites already enable that, like Shift MS, for example, and maybe the MS Society’s website itself, I don’t know it well enough. So having that facility for people to hook up in some way, communicate with each other in some way is probably a good thing. [P2 – CG2]*  *I think if it was just for people who were accessing the course [F: yes, that’s who it would be for] and with MS [F: it would be closed] [P3 –CG3]*  *There’s a lot of MS groups where you can do that now anyway. Where you can pose a question as part of a community, Facebook pages and stuff. But it’s not dealing with a specific issue, activity. [P6 – CG1]* |
| **Group aspect / Webinar** | *P7: I think we should provide that option; that flexibility - if they want to say something about themselves they could either video record themselves, prepare a slide and upload it whatever they prefer or not to say anything P4: But if that’s the interactive live bit, peer support bit that sits around this then that’s where you would do that bit  P7: But it doesn’t have to be live it could be pre-recorded  [Roundtable]*  *…there’s something different that happens when you’re physically in the room with someone. Even seeing their face online is not the same. [P5 – CG1]*  *And I thought, actually, the length of time was important. You know, there was very much a difference between people beginning of the programme to end of the programme. So that, building one week on another on another, in terms of the dynamic in the group. And I think it’s important to keep it a closed group. You know, it was the same group that started and the same group that carried all the way through. Rather than people coming in, was a key aspect for us.[HCP 5]*  *When we met here, you talked to people about other things besides MS. Real, rounded people. If you could do that. If you could encourage them to get to know each of the other members individually as real people, that would facilitate working together. [P1 – CG1]*  *It’s a lot of the soft stuff that you miss on a webinar. If you were sitting in front of a camera, you’d be, as you said, it’s this bit you’d get (pointing to upper body) and you’re probably straight looking at the camera. You wouldn’t get all the other body, the hands, what I’m doing now. [P4 – CG1]*  *I think that is the biggest challenge transferring it from a group session. Because for me that was the biggest thing that I got out of it. [P3 – CG2]*  *And it would be really good if you could, if you’re setting a few people off on the programme, they can log in to that at any time during the week, but have the opportunity to come together on a skype link or some such method. ‘Cause I think the group element of it is something that really, people really benefit from. Because they hear stories, I always say a lot of the information that I got, that I’ve shared with my client group is gained from people with the condition. Because they know what it’s like. Lots of the tips and things I share come from other people. I just, recirculate them. Not everything, obviously, but you know, that’s a really good way of sharing information, isn’t it? So I think it’s really important that that’s not lost if it’s at all possible to keep it.[HCP 7)*  *I personally have taken part in a number of online courses myself, and like the big interaction, like having a video to watch, and then like either a question to answer or a forum or something to engage in, so you can put your own answer on and obviously look at other people’s answers and then ask them questions and engage backwards and forwards that way. So I would envision something like that working quite well. [HCP 2]*  *I think doing the live aspects might be more challenging, um, from a health care perspective. And also just in terms of technology and stuff like that. ‘Cause you said about Skype and other bits and pieces and I, I have obviously used Skype and Google Hangout and things like that and they are, temperamental at best. And there’s nothing more, than you know, being frustrated at sitting there trying to take part in an online thing that is just not working. I’m still not convinced that we’re, especially within the NHS, up to the level of technology that would be needed to support that. [HCP 2]* |
| **Trust and safeguarding** | *Trust is a huge thing. I was only last week, drinking coffee after gym with a good friend of mine that I got to know over the time we’ve been attending the gym. My friend [XX] talking to me about how [description of MS symptom]. You’re not going to have those conversations with anyone apart from those of us who understand what’s going on. [P3 – CG1]*  *P5: I won’t trust them. I won’t trust them ‘cause I don’t know them. They’re not real, so. Personally, I don’t respond well to avatars. P2: Yeah, see I’m exactly the same. I think for something like this, you need that personal touch. I don’t think an avatar would give that. [CG1]*  *I must admit, I think that’s absolutely critical. Having that first session with people so you meet the people… so you have an informal discussion and you gain a bit of trust with the rest of the group. Otherwise, it will always be something that’s distant, remote, you are on a computer screen, I can switch you off, it’s not, there’s no personal contact, there’s no link to it.* *[P4 – CG1]*  *It’s a safe place. When you’re all together and expressing yourself, it’s a safe place. [P7 – CG1]*  *I guess they would deal with all the safeguarding issues with things that might come up….Sometimes I have been in groups where people have disclosed quite significant things in week 3/4 as the groups starts to form and that’s ok, ‘cause we can deal with that* —*‘cause we’re there it’s ok ….but I am just thinking if I was doing a session live online and something came up is that a [NHS] Trust safeguarding [policy] that I am following? [P1 - Roundtable]* |
| **Booster Sessions** | *Most of the people that we worked with have loved it so much that they ask if we could just keep doing it. Which is really sweet. And what would be nice would be able to say to people, there are booster sessions and you can go back in and you can access these freely. ‘Cause some people are looking for group involvement, other people are really looking at just the resources themselves. And looking at them regularly, so that would be quite useful. [HCP 4]*  *I think there is loads of scope which I haven’t thought of before, about the booster sessions. And having met the group and then doing the booster session online, that would be much easier for the therapist. ‘Cause you have at least got them in your head then. And you could do that rather than having to bring people back. So for your time and their time, ‘cause it does take some organisation with rooms and letters and all the rest of it. So that would be really good and I hadn’t really thought about that before we had this conversation. [HCP 3]*  *What we have done is, have used that force field sheet, at the very back in Session 6 and we’ve asked them to re-visit that and look at how many of those things did they find they applied and if they couldn’t remember some of the things, then we go back over it and see if we need to review. So, for example, some of them, when you’re doing that review, you might discover that quite a few of them have issues with sleep, or something, so then you might do a booster session on sleep. That’s what we have done. I got them to pick which of the things they did apply, which of the things that hinder that would still be an issue for them, and then we focused on that. [HCP 5]*  *I just wonder if maybe an actual follow-up might be useful for people as well, to have following the 6 weeks. Having a little break and then perhaps having an opportunity to come back together. But I suppose if there’s a forum, people can, and they’re used to using that, people can dip in and out, and if they can access that in the long-term, then hopefully that can keep the momentum going a bit as well. [HCP 6]*  *P1: A refresher, even just one day, one afternoon refresher. P4: yeah.  P2: That’s how online could help.  [CG2]* |
