# Supplementary material for: Digitizing a Face-to-Face Group Fatigue Management Program: Exploring the Views of People With Multiple Sclerosis and Health Care Professionals Via Consultation Groups and Interviews
Source: JMIR Form Res. 2019 May 22;3(2):e10951. doi: 10.2196/10951 (PMC6549474; doi:10.2196/10951)
Supplement: Multimedia Appendix 5 [file formative_v3i2e10951_app5.docx]

### Appendix 5: cFACETS Engagement Comments

Comments relevant to the engagement of users with cFACETS

| **Category** | **Example responses** |
| --- | --- |
| **Keeping people engaged or adhering to it** | *P1: It’s what you get out of it, isn’t it? If you think, ‘I’m benefitting from this’, then you’ll want to do it. If you think ‘it’s a waste of time’ then you’re not going to bother. P4: Yeah, you’re exactly right.  [CG2]*  *What about at the beginning before you apply or go on it have sort of .… somebody talking like a group first of all to say “this course has helped” so have that as a punchline …..So say “After doing this FACETS group….” or whatever with little captions or something I don’t know but a group and then you log on to it so that you think oh ‘I’ll have a go of that because these people are normal and they are human beings and they have done it and they are talking about it’. I think that attracts you [P3 – CG3]*  *I think one of the week’s goal-setting is part of the week’s topics. So actually having some way to actually set those goals and then, I guess one of the things that we don’t really see within FACETS too much, is people reviewing those goals. ‘Cause they could be quite long terms goals. And if there was some way that they could continue to engage with the process and tick off when they feel they’ve achieved a goal or something, that would be quite nice. If it flagged up a little “ping” back to your therapist or go look, ‘I attended this course, I did it, 6 months ago, but I achieved my goal’, that would be quite nice. [HCP 2]*  *For instance, I’m sort of obsessed with learning languages online, and so I use a couple of apps and things. And I was thinking why do I keep going back into some and not others. And I think it’s because they’re bite-size, they’re small enough to dip in and out of when I need to. If I’ve got some time on my hands, I can pop into it. [HCP 4]*  *I think the challenge would be to get people to carry it through completely. You talk about pausing. Some people pause it, ‘Oh I’ll go and make a cup of coffee’ and then suddenly the phone will ring…. [P1 – CG1]*  *You’ve gotta have something that …you’ve gotta look at it ‘cause you’ve got all these apps and everything that say ‘this’ll help you ..don’t eat this, don’t eat that’. You’ve gotta have something that goes, ‘we’ll tell you that this was really interesting, there’s feedback on there to say that people have gone on that course and it’s changed their lives’. “You know might not be for me but I’ll give it a go.” You know and they make that decision at the end of it, don’t they? [P4 – CG3]* |
| **Keeping in touch / reminders** | *But so, on the homework tasks, somehow they’d get some feedback on their progress to get encouragement or some troubleshooting. I don’t know how you do it, but something. [HCP 1]*  *F: OK would you like any reminders, automatic reminders to ask you how you are getting on you could opt in? P4: I’d like a reminder. F: You could get an email or a text message P4: Reminders I like…  [CG3]*  *But I think you need to make them personal. You know, “if you decided to leave the programme that’s ok, but just wanted to check you’re alright” and, you know ‘cause that’s what we would do here. But we’re also quite strict with people. So we do tell people at the beginning, that if they miss 2 [sessions] then they get put on the top of the waiting list for the next group. So that they can’t, come to week 1, miss week 2, come to week 3, miss week 4, then come to….. you know. There’s got to be a commitment there. [HCP 3]*  *There may be some people who don’t want, who are more individualistic and would want to do it on their own. Actually, I can think of one or two and there are others who, just by their nature, are more kind of social and like the idea of, you know, six of us are starting together, and yet we can do it from the comfort of our own home. And we’ve one facilitator who will touch base with us at the beginning, at the middle, at the end, or something. So there’s maybe different models for doing it. And probably it would serve both. [HCP 5]*  *[asked about use of reminders] I think that could be definitely helpful. But I think that sometimes people, once they first have been on something, sometimes momentum might slip. But if they are struggling, sometimes people don’t always remember to go and do what they first found helpful. So it might be useful to just remind them that there would be that facility there for them. [HCP 6]* |
| **Homework** | *Well, maybe there needs to be the app on their phone that goes with it. And they can just easily enter something into a Smartphone or whatever they’ve got, just like your Fitbit buzzes at you at 10 to the hour, probably mine ...Something that just buzzes and says ‘complete your…. what are you doing now?’ Or something. I don’t know. I think a prompt in an app saying that ... I don’t know. Maybe you can do it then for the rests. ‘Have you, are you taking your rests?’ Or ‘Have you?..’. I don’t think it all has to be that, but the activity diary, you almost need a prompt, don’t you? But if you can enter something on your phone easily. [HCP 1]*  *Everywhere I go, I’ve got my phone. If I’ve got a few minutes, I sit and fill it in and if it’s fresh and current, I wouldn’t fill in paperwork. Even on the course, I’d fill in the paperwork the night before, or the morning I was coming to the class. But if I had it on my phone, I’d be more inclined to fill it in. [P4 – CG1]*  *The more you put into your homework, the more you’ll get out of the course as a whole. And over time, there’s always the odd one that hasn’t done stuff, but by the end of the group, even if people haven’t written things down, they’ve been thinking about it during the week ‘cause they know there isn’t the pressure to write it down. And I don’t know how you would do that online. [HCP 3]*  *Because that was very helpful, writing. I think you can read something or listen to something but when the homework came you actually unpicked it …your own …where you are. [P4: Yeah] and the way you needed to change your lifestyle which you know if you didn’t have the homework I don’t know whether – you wouldn’t really…you could read it and think oh yeah I do that but then when you actually with your homework you’re actually writing down your planning and your you know sort of we did an exercise where we had tasks and ‘Do we do it now? Do we do it later? Do we delegate?...’ and that sort of thing you can read that but when you actually do it you think, “Oh yeah”, you know. “That’s what I do, but hang on a minute, maybe I could do that…” But I think that, for me anyway, I need to see it. I need to write it down to understand and act on. [P3 – CG3]  I think the homework that you set… you have got to be really careful that it’s not difficult or challenging to understand. ‘Cause when you have got the person there who explains what it is about and the reasons for stuff and not to be critical of your course but I can remember the… well, [XX] saying sometimes, “Don’t worry about.…” I think there was a scaling one where you had to measure and she said, “Well a lot of people have had trouble with this in the past, so don’t really worry about it, just think about this and that.” [P2 –CG3].*  *Normally in the session before you get the homework, it’s all revised and you go through it so you know what’s in there. So like you said, even if you don’t do the homework, they’ll know what the homework’s about, they’d probably have in their head how they would have done something. Or they may have thought, I haven’t written that down yet. But it’s actually Wednesday so I know I can’t be bothered to fill it in. You all, I know that week that I did this on a Monday and that on a Friday, but I didn’t actually write it in. But you’re still thinking about it. [P6 – CG1]* |
| **Introducing cognitive behavioural model online** | *Well, I don’t know whether it’s an overt thing that you do or whether it’s, ‘cause the FACETS group is quite nice in that by the time they actually bring up the model, the person is actually familiar with all the terminology because they’ve heard it in every week beforehand. So that’s been talked about the whole time, but they haven’t, it hasn’t been called that until, I think it is the 5^th^ session. So, when it finally does come up, people are really open to it because they’ve heard it, they’ve worked with it, they understand it, it makes sense to them.[HCP 4]*  *It’s quite, there’s quite a lot there. I think. I don’t know how you’d do it, but I think you’d have to do it very gradually and lots of examples. So, maybe the way you do it, something comes up and you have to, I don’t know how you’d do it actually. Something will come up on screen and they would apply each stage, you know, they’d apply it to themselves and the next stage, just do it very gradually. [HCP 1]*  *I think online would be quite a good place to introduce it actually. Because obviously you’ve got the model, which is quite a flat thing on a PowerPoint. But you can have clickable sections, where you can kind of click on it and see what it said about, you know, the impact of the environment, the impact of thoughts, feelings, etc., etc. So I think that could be actually quite a good way to introduce that. …….I think if people had a chance to really interact with it and kind of get to grips with understanding it, it might help them, kind of make better links between their own environments and behaviours and thoughts, etc. [HCP 2]*  *I suppose you could use slides. I suppose a video makes it more personal though….. I would probably prefer the talk; the video, with giving some examples as well. [HCP 6]*  *I think that really, we played the Bingo today we put them, we only went through it last week, we put the model up, they were like, ‘I don’t think I’ve seen that’ – we showed it to you last week, we talked about it quite a lot. That model to them is meaningless but they remembered all the different negative styles of thinking. That is what they remembered. ‘I’ve never seen that’ and we were like, ‘we’ve definitely done it last week I promise’. So yeah, I think that is a really difficult model for them. I suppose if there’s any way of making it interactive where’d you click on it and have someone explaining what that means, rather than them having to read about it. [HCP 8]* |
| **Rewards /Gamification/ Goal Setting** | *So even with the goal setting. They needed a bit of, very few were able to just go away and do that on their own. They needed to think about an idea and have some support of talking it through before they really crystallized it. And, without the goal setting, I think it’s hard. I think people are less likely to actually maybe implement it. [HCP 5]*  *I suppose if there’s any way of linking it to the Smartphone I think that would help people to be engaged as well. Even if it’s like as simple as like a… week 1 a massive tick on your little participation chart or like a little trophy or something to keep them involved. [HCP 8]*  *Maybe showing some evidence that a positive change can result in meeting your goals. To make sure your goals comply with whatever criteria and SMART is one thing, but the actual content itself is key. [P2 – CG2]*  *WP1: You could have examples. Different examples for different people because we all have our SMART goals for different areas. So just give an example and get you to think about a specific area of your life you would like to apply the SMART goal to F: So having some examples to help explain it? P1: Yeah, a number of different types F: And people talking about the goals they have set? P3: Yeah  F: And maybe reassuring people - don’t worry if you don’t meet them as you were saying…  [CG3]*  *I guess if, if people, if you think about goal setting and SMART goals and they set something for themselves, and that what’s their task was when that comes….and there is some way of them feeding back if they did, honestly, ‘yes, I did go do my swimming’ or ‘I didn’t’. Um, that, I think that, and then it’s been acknowledged in some way, I don’t know how. I think that would be encouragement. That’s what you get from the group. That’s what encourages you to do it. I think if there’s no feedback, or no opportunity to say whether you’ve done it or not, I know you can always lie anyway and say you have and you haven’t. But, the fact is, you know you’re lying to yourself. Whereas if there’s no requirement to give any feedback, then, you’ll just skip it, I think. [HCP 1]*  *It doesn’t matter if you don’t make them [goals] you can always try again as it all depends on how you feel doesn’t it, on that day. There are plenty of times when I haven’t achieved it and I thought well I’ll make sure I try and get that but it’s how you feel when you get up that day isn’t it? So there is no right way and no wrong way. It’s what suits everyone. [P4 – CG3]*  *I haven’t explored gamification that much. But you know, I’ve downloaded a couple of apps myself that kind of have little gaming aspects, I guess. You could have a little individual with fatigue and you could try and give them advice and see if their fatigue gets better or not. I don’t know! [HCP 2]*  *Most people that I’ve run the programme with have found it really, really beneficial. We’ve had lots of really good feedback. We haven’t had any negative feedback. But we’ve had lots of really good feedback. And kind of objectively I think, it’s because they’ve found the rewards of implementing the strategies. Not because we’ve provided them with any incentive to complete the course, or anything like that. [HCP 6]* |
| **Progress bar / dashboard** | *Well, their progress through the course. So like when you do one of those online surveys and they say, you still have 80% to do? I don’t know. I wonder whether at the beginning if that could be off-putting. If you think you’ve only completed, you know, one sixth of it… [HCP 1]*  *I guess some sort of check-in to say that they’ve read the material would be useful. So I know some courses have some, like a monitor, so as you go through and watch each video clip or anything, it kind of just ticks that you’ve done it and then you get a progress bar, I guess, that says how much of the information you’ve read and looked through, or interacted with [HCP 2]*  *[Talking about the Duolingo app] And you start through it and so you have, the first programme you go into probably has 4 different elements to it and then at the end it trumpets, “ Well done, you’ve got to the end of session 1!” And I think it gives you Duolingo points or something. But that doesn’t interest me as much. But it’s really great to get the trumpet sound. Oh great, I completed that! That’s great. [HCP 7]*  *I would gear it so they have to, it’s a reward isn’t it? They have to submit an activity diary. They have to submit a plan. They have to sort of get some feedback on resting or, it doesn’t have to be big, but just to have some ownership of it. Before they, and that unlocks...and also if they see the whole lot, they know what’s coming ahead. So they just jump ahead in their mind. So I think I like the idea of unlocking something when you show, even if it’s just your cursory, you’ve done something, you’ve ticked to say you’ve completed it. [HCP 1]*  *[talking about the dashboard concept] …that’s a good idea. It shows you how far you’ve gone and, you know, anything that goes “Congratulations, you’re now halfway through” [HCP 5]*  *P7: It is useful to have that, how far you’ve gone.  P6: You might have people feeling that they can’t proceed any further if they haven’t got everything ticked…  [CG1]* |
