# Supplementary material for: Digitizing a Face-to-Face Group Fatigue Management Program: Exploring the Views of People With Multiple Sclerosis and Health Care Professionals Via Consultation Groups and Interviews
Source: JMIR Form Res. 2019 May 22;3(2):e10951. doi: 10.2196/10951 (PMC6549474; doi:10.2196/10951)
Supplement: Multimedia Appendix 6 [file formative_v3i2e10951_app6.docx]

### Appendix 6: cFACETS Interactivity Comments

Comments relevant to the interactivity of cFACETS

| **Category** | **Example responses** |
| --- | --- |
| **Flipcharts** | *I think so, but as much to stimulate ideas. I think it’s, you know, you write things up and it’s a visual cue I think, when people look at them. I think it’s, obviously at the beginning we do, what their aims are, and looking at that at the end, I think that’s useful, to reflect back. But the flipcharts are really short-term. They’re quite, of the moment. [HCP 1]*  *What came through quite strongly is that they are fluid and link things together in the group, good to stimulate ideas and somebody says there seems to be a bit of repetition but actually I think that bit of repetition is intentional because we’re trying to…..it’s doesn’t matter….get the message across…if there’s a bit of repetition and overlap because you’re building on each week so personally I don’t think that’s necessarily a bad thing. [P3 - Roundtable]*  *…the danger with that is then it becomes quite scripted then, doesn’t it? Whereas, if you were putting up what your group has said, there’s much more ownership of it. So in FACETS now, you do a flipchart and then you show a PowerPoint don’t you, this is what possible ideas have come up. And, we then check off what’s on the PowerPoint was what we’ve got on the flipchart. And very often in groups, people say, “oh we’ve done very well, we’ve got all of them.” Whereas, if you were bringing stuff up, it would be quite formulaic then, wouldn’t it? [HCP 3]*  *But with the flipchart, we sort of put up the question and then if you’ve got a really quiet group, then the therapist will say, “hmm, when we have run groups before, perhaps somebody might have said this” –and then write it up, and that would get people talking. Or somebody would say, “no I don’t agree with that” or, you can, it’s much more fluid isn’t it? … And it kind of links things together. I very often find that , that I’m drawing one comment and drawing a line between that and another to link ideas together for people, so that they can see it. [HCP 3]*  *The one that people seem to really love when we run the group, they like most of the flipcharts, but the one that people really love is the one around stress. It’s about coming up with symptoms for themselves against the body. To me, that’s a really powerful part of the course is understanding that perspective, stress mechanisms versus performance and how people feel and how it affects them doing things. So, when we do that body shape and list the symptoms up or feelings and bodily sensations, people find that really helpful, like a real eye opener for people. [HCP 4]*  *We use flipcharts a lot actually, and I do find them really helpful. And I think it’s helpful for the group as well, because they quite like to see it written up there and when they’re trying to generate their own ideas, seeing someone else’s ideas can help facilitate it. So I guess, there might be creative way of doing that online as well where, they might put some words up and when they contribute maybe a few other ideas come up that others had suggested and then they can suggest another one. [HCP 4]* |
| **Technical Interactivity** | *…so something, some visualisation and deep breathing, some way that you have a sort of little video that would play or I don’t know enough about online things, but or something like a voice coming through that would tell them. Just like I would do it at the end, you know, once a session, visualisation or deep breathing. They have a voice delivering exactly the same thing. But different ones and they can find what works for them. I think that would be really important. To have the relaxation delivered. [HCP 1]*  *So I think with the tasks, just having more tasks, more click buttons, more things that they have to fill in so they’re actually getting a bit more ownership of it. [HCP 1]*  *Yeah. Videos are much easier to watch than to read the word. [P5 – CG1]*  *[About a written transcript]*  *Now I know that’s not the same in FACETS, but it just means that if somebody hadn’t absorbed it because they had problems with information processing, which we know people do, don’t we, in MS. Then, that might be really helpful for them, it would give them a different media to, they’d be able to process written information rather than auditory. [HCP 3]*  *[About videos of pwMS]*  *…even if you couldn’t have the conversation, the one thing I was just thinking about was maybe see people that you’ve previously done the group with and have them have a chat with each other, like they’re doing the group again. And have that sort of chat, like as if we’ve just done an exercise. Me and [XX] could talk to each other, “Oh how did you get on with that?” That could then be fed back to them… [P2 – CG1]*  *I’m wondering if there’d be any potential to have a video of a group speaking about different things….the way [XX] has just spoken about …that’s very very powerful [P2 – CG3]*  *And I think, because what you’re getting from an online programme is you’re getting the professional information. But you’re not getting the lived experience. If the video could capture somebody’s lived experience. Or even somebody who’s done the programme who says look, “Before, this is what I found fatiguing and these are the strategies I put into place, and this is how it helped.” You know, just something to capture somebody’s lived experience. [HCP 5]* |
| **Real or virtual** | *[About existing examples of animations shown to the group]*  *Something like that you can watch it without cringing. Whereas the other one, you see them….. I know it was only an example. But something like that you go, maybe not. But that one was, it was factual without being heavy, but also without the cartoony bit. It isn’t necessary all the time. [P3 – CG3]*  *F: Like a cartoon character P2: I wouldn’t like that- P5: I won’t trust them. I won’t trust them ‘cause I don’t know them. They’re not real, so. Personally, I don’t respond well to avatars. P2: Yeah, see I’m exactly the same. I think for something like this, you need that personal touch. I don’t think an avatar would give that. [CG1]*  *The thing is, when you’re looking maybe at the memory and things like that. You’ve got a psychologist or something else, something like that, and you actually name them and they’re presenting, that gives a bit more credibility. And then perhaps an occupational therapist doing some other aspect. [P3 – CG2]*  *Yeah, ‘cause potentially you’ll upset people there where they’re thinking, well, is my illness real or is there someone, and again I will use a word that might upset people, is this real or is this someone trying to take the p*** out of me... [P2 – CG1]* |
