# Supplementary material for: Digitizing a Face-to-Face Group Fatigue Management Program: Exploring the Views of People With Multiple Sclerosis and Health Care Professionals Via Consultation Groups and Interviews
Source: JMIR Form Res. 2019 May 22;3(2):e10951. doi: 10.2196/10951 (PMC6549474; doi:10.2196/10951)
Supplement: Multimedia Appendix 7 [file formative_v3i2e10951_app7.docx]

### Appendix 7: cFACETS HCP Relationships Comments

Comments relevant to the relationships HCPs might have with cFACETS

| **Category** | **Example responses** |
| --- | --- |
| **HCP Involvement** | *What’s becoming more tricky is, the way that the NHS is going, is that most of our time is really finely tuned in terms of the resource of it. So, to get time out of clinical activity, and face-to-face clinical activity can be harder and harder to do. And particularly for hospital [NHS]Trusts. I don’t know so much about the community [setting], but I imagine they’d have the same issue… [HCP 4]*  *I think it would massively vary, I know for a fact that if I was involved in it I would agree to sign up to do a time. I mean if it, if it’s more of a national thing and you have a list of facilitators who run the course who’d be happy to share it amongst everyone then really it might only be like a 2 hour commitment twice a year or something like that. It would reduce it dramatically. If everybody’s going from the same script still, so someone has the same information, then you’d be giving the same information to the patients. So that wouldn’t, you wouldn’t be so concerned about consistency but I suppose if it was just the local therapist having to do that then I think it would be a problem definitely. [HCP 8]*  *…but I don’t think, you know, for example, I work 16 and a half hours a week, my colleague works 12 hours a week, we won’t have capacity to kind of look at and respond to everything. We’d probably do it more generally in a group environment, I think. [HCP 2]* |
| **Supporting healthcare workers / complementing care** | *…being able to either look at their progress might be good, or depending on what they wanted, they could allow us access to see all their responses and stuff as well. It could be stuff that they might want to do, some of it privately, there might be a button that says, ‘send this information to my therapist’ . [HCP 2]*  *What that would be like if we were in fatigue clinic and could say, you could go to either a group or you could do this electronically. I think we might have quite a take up for that [electronically] because I think people are quite apprehensive about coming to a group. [HCP 3]*  *…there actually might be some healthcare professionals who’d be interested in actually doing the online course themselves. Just to get the information. So as a kind of a training tool. And, a lot of people, and I’m just thinking of community colleagues who may not have a whole cohort of people with MS, but only have about one out of their case load. It’s a way of having a bit more of an expert input. [HCP 5]*  *When somebody’s embarking on something, you want to give them as much support and encouragement as possible. So I don’t know. You may be looking at two completely different programmes. One that offers more of a baseline information that they can access without any healthcare professional support. And one that is a true online version that you can support people with going through. [HCP 7]*  *It won’t have the same effect because you’re not in a group but if you could sort of bottle that and put it in an online I think people will benefit it from it. But I do think for me I just think I just loved the course. But I think it’s fair to say when people haven’t got the access or disabilities and they can’t get there or maybe someone is a recluse and doesn’t want to be part of a group then it’s a really good option to have that facility online as well. If they can implement that near enough to the same as the group. [P4 – CG3]* |
